# Cyberlindnera hibernica sp. nov. and Barnettozyma discipulorum sp. nov., isolated from forest soil in Ireland

**DOI:** 10.1099/ijsem.0.006898

**Published:** 2025-09-09

**Authors:** Adam P. Ryan, Cláudia Carvalho, Yiran Zhao, Julianna Decuseara, Matthieu Osborne, Padraic G. Heneghan, Kevin P. Byrne, Tadhg Ó Cróinín, Kenneth H. Wolfe, José Paulo Sampaio, Geraldine Butler

**Affiliations:** 1 School of Biomolecular and Biomedical Science, Conway Institute, University College Dublin, Belfield, Dublin 4, Ireland; 2 PYCC - Portuguese Yeast Culture Collection, UCIBIO, i4HB, Departamento de Ciências da Vida, Faculdade de Ciências e Tecnologia, Universidade Nova de Lisboa, 2829-516 Caparica, Portugal; 3 School of Medicine, Conway Institute, University College Dublin, Belfield, D04 V1W8 Dublin 4, Ireland

**Keywords:** chromosomes, genome assembly, Illumina sequencing, nanopore sequencing, *Phaffomycetales*, phylogenomics, yeast

## Abstract

Two yeast strains, PYCC 10015 and PYCC 10016, were isolated from soil from an Irish forest. Sequence analysis of the internal transcribed spacer (ITS) region (ITS1-5.8S-ITS2) of the rRNA gene repeat, and the D1/D2 domain of the LSU rRNA gene, showed that they belong to the *Cyberlindnera* and *Barnettozyma* genera of the order *Phaffomycetales*, but they did not exactly match any known species. The genomes of both isolates were sequenced using Oxford Nanopore Technologies and Illumina sequencing, generating chromosome-level genome assemblies. Phylogenomic analysis of 1,385 single-copy orthologues from 37 *Phaffomycetales* species and 2 outgroups showed that the closest relative of PYCC 10015 is *Cyberlindnera galapagoensis* and that PYCC 10016 is placed in a subclade containing 7 other species from the *Barnettozyma* genus. The average nucleotide identity between these strains and their closest relatives is <75%, supporting their designation as novel species. Here, we propose the names *Cyberlindnera hibernica* sp. nov. and *Barnettozyma discipulorum* sp. nov. for PYCC 10015 and PYCC 10016, respectively.

## Data Summary

The internal transcribed spacer sequences (PQ384455 and PQ384456) and the D1/D2 domains of the LSU rRNA gene (PQ373936 and PQ373935) of *Cyberlindnera hibernica* PYCC 10015 and *Barnettozyma discipulorum* PYCC 10016 are available at the NCBI. Chromosome-level assemblies of strains *C. hibernica* PYCC 10015 and *B. discipulorum* PYCC 10016 can be found under BioProject PRJNA1167496. Genome annotation for these species is available on FigShare at the following DOI: https://doi.org/10.6084/m9.figshare.27838869.

## Introduction

*Barnettozyma* and *Cyberlindnera* are two genera within the order *Phaffomycetales*, containing 11 and 31 described species, respectively [1,2]. Species of these genera were initially placed in the genera *Pichia*, *Issatchenkia* and *Williopsis* based on phenotypic similarities*,* but following molecular phylogenetic analysis by Kurtzman *et al*. [3], they were reassigned to newly proposed genera *Barnettozyma* and *Lindnera*. The name *Lindnera* was later replaced by *Cyberlindnera* due to a homonymous pre-existing plant taxon [4].

*Cyberlindnera* species are variable in habitat, but many are found in forest biomes in association with rotting wood [5,9]. Some are opportunistic pathogens such as *Cyberlindnera fabianii*, which can form antifungal-resistant biofilms and cause invasive bloodstream infections [10]. *Barnettozyma* species are also commonly found in decaying wood and plant matter [11,15].

*Cyberlindnera* species are known to ferment various sugars apart from glucose, including inulin, raffinose and sucrose [7,16, 17]. Both genera *Cyberlindnera* and *Barnettozyma* are of industrial interest because several species can metabolize xylose from lignocellulose biomass [1,13, 16, 18]. This can be exploited in the production of xylitol, a sugar substitute with various food and pharmaceutical applications [16,18]. Known species from both genera do not assimilate hexadecane or methanol [3].

The *Cyberlindnera* genus contains both homothallic and heterothallic species, which form Saturn-shaped and hat-shaped ascospores, respectively [5,8, 19]. Homothallic and heterothallic species of *Barnettozyma* have also been described with Saturn-shaped and hat-shaped ascospores, but it is not clear if ascospore shape is correlated with thallism in this genus [3,11].

In this study, two yeast strains PYCC 10015 and PYCC 10016 were isolated from forest soil samples collected in 2023 as part of undergraduate research projects conducted at the University College Dublin, Ireland. Divergence in both internal transcribed spacer (ITS) sequence and the D1/D2 domain of the LSU rRNA gene suggests that PYCC 10015 and PYCC 10016 represent novel species belonging to the *Cyberlindnera* and *Barnettozyma* genera, respectively, hereby proposed as *Cyberlindnera hibernica* and *Barnettozyma discipulorum*.

## Methods

### Isolation, cultivation and identification

Yeast strains were isolated from soil as described in Ryan *et al*. [20] and Bergin *et al*. [21]. Soil samples (~2 g each) were taken, using a clean trowel, from the bases of ten mature Irish sessile oak (*Quercus petraea*) trees at Glengarriff Woods Nature Reserve, County Cork, Ireland. The dominant habitat in this nature reserve is old oceanic sessile oak woodland. One sample was taken per tree, at a depth of ~3 cm below the surface, avoiding leaf litter. Glengarriff Woods is public land owned by the Irish state and is managed by the Irish National Parks and Wildlife Service (NPWS), which does not require a permit for collecting micro-organisms.

In brief, ~0.5 g of soil was incubated in 9 ml yeast-peptone-dextrose (YPD) broth (1% yeast extract, 2% peptone and 2% glucose) containing antibiotics (chloramphenicol 30 µg ml^−1^ and ampicillin 100 µg ml^−1^) for 5 days at room temperature. These cultures were then mixed to homogeneity, and 10 µl of each was inoculated into fresh media for a further 2-day incubation at room temperature. To enrich for yeast species, dilutions were plated onto YPD agar (1% yeast extract, 2% peptone, 2% agar and 2% glucose) and incubated for a further 5 days at room temperature [22]. Plates containing large numbers of filamentous fungal colonies were discarded. Single colonies of potential yeast species were isolated for further investigation.

Sequence analysis of the ITS region (ITS1-5.8S-ITS2) of the rRNA gene repeat and the D1/D2 domain of the LSU rRNA gene was used for species identification. The ITS sequence of each yeast isolate was amplified from single colonies using primers ITS1 (5′-TCCGTAGGTGAACCTGCGG-3′) and ITS4 (5′-TCCTCCGCTTATTGATATGC-3′) [23]. D1/D2 sequences were amplified from cultures using primers NL1 (5′-GCATATCAATAAGCGGAGGAA-3′) and NL4 (5′-GGTCCGTGTTTCAAGACGG-3′) [24]. Amplicons were sequenced with the Eurofins Genomics Mix2Seq platform using ITS1 and NL1 as primers. blast [25] was used to compare these sequences to known species included in the NCBI GenBank database [26]. Two isolates, PYCC 10015 and PYCC 10016, lacked conclusive matches to known species in the GenBank database and were selected for further characterization. Phylogenetic analysis of ITS and D1/D2 sequences was done using the Seaview package (v. 5.0.4; [27]), with sequence alignment by muscle and tree construction by maximum likelihood (PhyML with the general-time reversible substitution model), using Seaview’s default parameters, with 100 bootstrap replicates.

### Genome sequencing and assembly

To characterize the genomes of PYCC 10015 and PYCC 10016, we sequenced their genomes using a combined Oxford Nanopore Technologies (ONT)/Illumina sequencing strategy. For short-read Illumina sequencing, genomic DNA was extracted using a phenol–chloroform–isoamyl alcohol method as described in Bergin *et al*. [28]. Illumina libraries were prepared using Illumina DNA prep (Catalogue ID: 20018704) and indexed using IDT for Illumina indexes (Catalogue ID: 20027213). Genomes were sequenced in paired-end format (2×150 bp) using an Illumina NextSeq 2000 instrument. This generated 2,968,118 and 3,446,513 read pairs for PYCC 10015 and PYCC 10016, respectively.

High molecular weight DNA for Nanopore sequencing was obtained using the MasterPure Yeast DNA Purification Kit (MPY80010; Biosearch Technologies) according to the manufacturer’s instructions. Sequencing libraries were prepared from 1 µg of purified high molecular weight DNA per isolate using the Native Barcoding Sequencing Kit (SQK-NBD114-24; ONT). Libraries were sequenced using r10.4.1 chemistry flowcells (FLO-MIN114) on a MinION MK1C (ONT) device with MinKnow v. 23.07.12. Basecalling was performed using the fast configuration at default settings.

Nanopore sequencing data were filtered by average read quality and read length using NanoFilt v. 2.3.0 (ONT). Reads were filtered to minimum mean qualities of 7 for both species. Reads were filtered to minimum lengths of 1,000 bases for PYCC 10015 and to 5,000 bases for PYCC 10016. Data from both genomes were assembled using Canu v. 2.2 [29] with default settings. Initial assemblies were polished with NextPolish v. 1.4.1 [30] using short-read sequencing data. Five rounds of polishing were performed. Assemblies were filtered to remove repeated and degenerated contigs. Genome annotation of PYCC 10015 and PYCC 10016 was performed for phylogenomic analysis using the Yeast Genome Annotation Pipeline (YGAP) [31].

### Phylogenomic analysis

The phylogeny of PYCC 10015 and PYCC 10016 and 37 other species was determined using a phylogenomic analysis. The 37 other species were chosen by reference to a recent phylogenomic analysis by Opulente *et al*. [2], and they include all the available sequenced genomes from species in the sections of the genera *Barnettozyma* and *Cyberlindnera* that appeared to be closest to PYCC 10015 and PYCC 10016 from initial blast and analysis of ITS and D1/D2 sequence data (Figs S1 and S2, available in the online Supplementary Material), as well as some other phylogenetic landmark species and two outgroups.

**Fig. 1.**
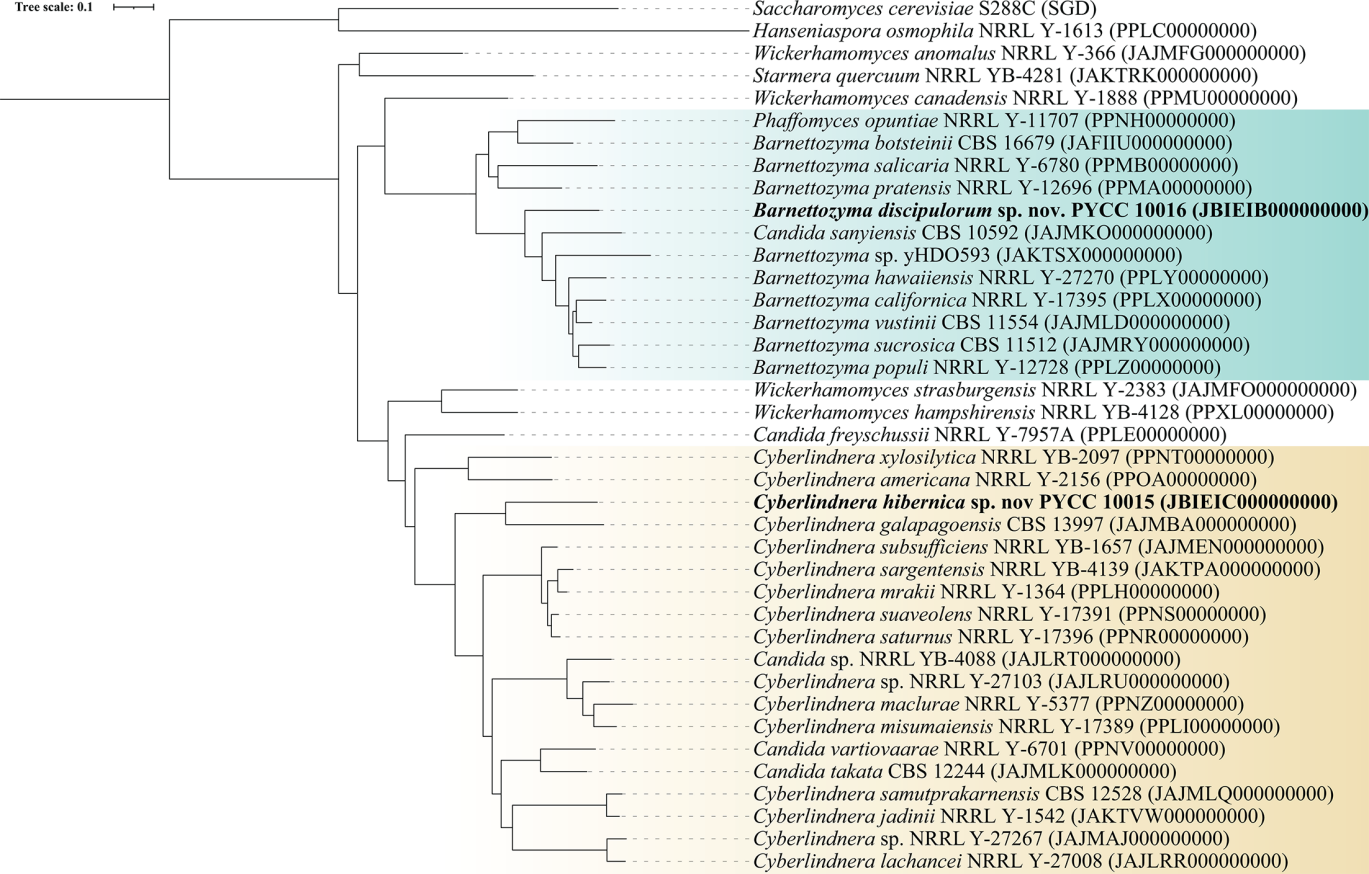
Phylogenomic tree created from 1,385 SCOs from 37 *Phaffomycetales* species and 2 outgroup species, *S. cerevisiae* S288C and *H. osmophila* NRRL Y-1613. Genome assembly accession numbers are shown in parentheses at each terminal node. The tree is rooted using the outgroup species. All bootstrap values are 100%. The *Barnettozyma* and *Cyberlindnera* clades are colored in blue and gold, respectively. Novel species *C. hibernica* sp. nov. PYCC 10015 and *B. discipulorum* sp. nov. PYCC 10016 are marked in bold text. The scale bar shows 0.1 aa substitutions per site.

The phylogeny of all 39 species was determined using an alignment of 1,385 single-copy orthologs (SCOs) as described in Ryan *et al*. [20]. In brief, short proteins (<100 aa) and highly degenerated sequences (>10 internal stop codons) were first removed from the predicted proteomes of all 39 species. Protein sequences were grouped using blastp v. 2.10.0 [25], retaining those with E-value ≤1×10^−10^, per cent identity ≥30% and per cent match length ≥70%. Orthologue clustering was performed using OrthoMCL [32] with an inflation parameter of 1.5. Clustering identified 1,407 putative SCOs (gene clusters with one sequence per species) in all 39 species. Alignments of each SCO were generated using MAFFT v. 7.520 [33] with parameters ‘--maxiterate 1000’ to run 1,000 iterations of alignment and ‘--genafpair’ to use the global pairwise alignment strategy. TrimAL v. 1.4.rev15 [34] was used to trim alignments using the ‘-gappyout’ parameter to remove highly gapped columns in alignment. Individual SCO trees were generated using RAxML v. 8.2.12 [35] with parameters ‘-N 5’ to specify the number of independent tree searches, ‘-m PROTGAMMAAUTO’ to automatically select the best substitution model and ‘-p 12345’ to set the seed. Individual SCO trees were rooted using the outgroup species *Saccharomyces cerevisiae* S288C and *Hanseniaspora osmophila* NRRL Y-1613 [2]. Tree topologies were automatically assessed using the ETE Toolkit in Python [36], and SCOs whose trees did not contain a branch separating a monophyletic *Phaffomycetales* from these two outgroups were discarded from further analysis. Trimmed alignments of the remaining 1,385 SCOs were concatenated, and the species-level phylogeny was calculated using RAxML [35] with parameters ‘-m PROTGAMMALG -N 5 -# 100 p 12345’, to set the substitution model to PROTGAMMALG, the number of independent tree searches to 5, the number of bootstraps to 100 and the seed to 12,345. The final bootstrapped tree (Fig. 1) was visualized in iTOL [37].

### Physiological characterization

Standard procedures were followed for phenotypic characterization [38]. Assimilation and fermentation tests were performed in liquid media. Fermentation was ascertained by gas formation in Durham tubes. Phase-contrast optics were used for microscopy.

## Results and discussion

### Sequence analysis of ITS and D1/D2 regions of rRNA genes

Among 106 yeast isolates from Irish soil samples that were collected during our undergraduate student module in 2023, sequence analysis of the ITS region of the rRNA gene array detected 42 known species, predominantly in the genera *Kazachstania*, *Saccharomyces*, *Barnettozyma* and *Hanseniaspora* (Table S1). Two yeast isolates, PYCC 10015 (original designation: UCD1070) and PYCC 10016 (original designation: UCD2008), were identified as members of the genera *Cyberlindnera* and *Barnettozyma*, respectively. However, their ITS and the D1/D2 sequences did not have 100% matches to any sequences in the GenBank database. Both of these isolates came from samples from the same forest, Glengarriff Woods in County Cork, Ireland. We investigated ten soil samples from Glengarriff Woods, each from the base of a different oak tree, and isolated a total of seven known yeast species from these samples (Table S1), in addition to PYCC 10015 and PYCC 10016. No other yeast species were isolated from the soil samples that yielded PYCC 10015 (soil sample GGW10) and PYCC 10016 (soil sample GGW4).

For PYCC 10015, phylogenetic analysis of the ITS and D1/D2 sequences indicated that this isolate is closely related to *Cyberlindnera qingyuanensis* [17] and *Cyberlindnera galapagoensis* [16], as well as having high sequence similarity to some database sequences that do not correspond to formally described species (Fig. S1). In blastn searches against the NCBI database, the ITS sequence of PYCC 10015 (PQ384455; 585 bp) had the highest similarity to that of *C. qingyuanensis* NYNU 223283^T^ with 14.6% divergence (85.4% identity and 93% coverage; 27 substitutions and 59 indels, excluding the regions bound by the PCR primers). Its D1/D2 sequence (PQ373936; 574 bp) had the highest similarity to that of *Cyberlindnera* sp. CHS-2017a with 5.6% divergence (94.4% identity and 98% coverage; 18 substitutions and 14 indels).

For PYCC 10016, phylogenetic analysis of the ITS and D1/D2 sequences indicated that it is closely related to several *Barnettozyma* species (Fig. S2). In blastn searches against the NCBI database, the ITS sequence of PYCC 10016 (PQ384456; 565 bp) had the highest similarity to that of *Barnettozyma vustinii* CBS 11554^T^ with 15.6% divergence (84.4% identity and 74% coverage; 30 substitutions and 100 indels), and its D1/D2 sequence (PQ373935; 584 bp) had the highest similarity to that of *Barnettozyma populi* NRRL Y-12728^T^ with 8.5% divergence (91.5% identity and 99% coverage; 15 substitutions and 38 indels).

The extent of sequence divergence from known species in the ITS and D1/D2 regions suggested that both PYCC 10015 and PYCC 10016 represent new species. They substantially exceed the levels of divergence between some accepted pairs of species in the same genera (for example*,* we calculate that there is only 2% divergence between the ITS sequences of *Cyberlindnera saturnus* and *Cyberlindnera subsufficiens* and only 3% divergence between the D1/D2 sequences of *B. vustinii* and *Barnettozyma xylosica*). However, because we have only one isolate of each potential new species, we also sequenced their whole genomes to clarify their relationships.

## Genome sequence comparisons and phylogenetic placement

To further characterize the isolates and investigate their relationship to known species, we used whole-genome sequencing using both Illumina and ONT to generate chromosome-level assemblies. Both assemblies consist of seven nuclear chromosomes and the mitochondrial genome (Table 1).

**Table 1.** Genome assembly statistics

Strain	No. of chromosomes	Genome size (bp)	N50 (bp)	% G+C
*C. hibernica* sp. nov. PYCC 10015	7 nuclear, 1 mtDNA	11,516,225	1,955,003	48.56
*B. discipulorum* sp. nov. PYCC 10016	7 nuclear, 1 mtDNA	11,658,228	1,671,570	43.28

To place PYCC 10015 and PYCC 10016 within the order *Phaffomycetales*, a phylogenomic analysis was performed using SCOs from them and 37 other genome sequence assemblies. The analyzed dataset included all the genome assemblies that were available for species that were suggested by phylogenetic analysis of the ITS or D1/D2 sequences to be close relatives of PYCC 10015 or PYCC 10016 (Figs S1 and S2). The phylogenomic tree (Fig. 1) shows that PYCC 10015 belongs to the genus *Cyberlindnera* and is sister to *C. galapagoensis* CBS 13997^T^; there is 100% bootstrap support for the clade containing these two species. PYCC 10016 lies within the *Barnettozyma* clade and is sister to a clade containing seven other species (*Candida sanyiensis* CBS 10592^T^, *Barnettozyma* sp. yHDO593, *Barnettozyma hawaiiensis* NRRL Y-27270^T^, *Barnettozyma californica* NRRL Y-17395^T^, *B. vustinii* CBS 11554^T^, *Barnettozyma sucrosica* CBS 11512^T^ and *B. populi* NRRL Y-12728^T^). There are bootstrap support values of 100% both for the branch defining this clade of eight species and for the branch separating PYCC 10016 from the other seven (Fig. 1).

For the species with sequenced genomes shown in Fig. 1, we investigated genomic similarity by calculating pairwise average nt identity (ANI) and average aa identity (AAI) values within the *Cyberlindnera* clade and within the *Barnettozyma* clade (Tables S2 and S3), using the programs OrthoANI v. 0.5.0 [39] for ANI and CompareM v. 0.1.2 (https://github.com/donovan-h-parks/CompareM) for AAI. In *Cyberlindnera*, PYCC 10015 has the highest genomic similarity values to *C. galapagoensis* CBS 13997^T^ (ANI 0.732 and AAI 0.692), which agrees with its position on the phylogenomic tree. These values are lower than the ANI and AAI values seen between several other pairs of species in the genus *Cyberlindnera* and similar to the mean interspecies values among the analysed *Cyberlindnera* species (Table S2). In *Barnettozyma*, the ANIs of PYCC 10016 to its seven sister species range from 0.718 to 0.746, and the AAIs range from 0.692 to 0.737, with the highest similarities being to *B. vustinii* CBS 11554^T^ (Table S3). These values are lower than those observed between several pairs of accepted *Barnettozyma* species, which can be as high as 0.840 (ANI) or 0.872 (AAI), and they are similar to the mean interspecies ANI and AAI values among this group of species. In our opinion, the ANI and AAI values calculated from the genome sequences of PYCC 10015 and PYCC 10016 further suggest that they belong to novel species.

The AAI values for PYCC 10015 and PYCC 10016 can be compared with the AAI values for *Saccharomycetales* species reported in a recent comprehensive analysis by Liu *et al*. [40], which used the same method to compute AAI. The highest AAI values we observed for the new *Phaffomycetales* isolates were 0.692 for PYCC 10015 versus *C. galapagoensis* and 0.737 for PYCC 10016 versus *B. vustinii* (Tables S2 and S3). Many pairs of *Saccharomycetales* species have AAI values that are higher than these: values higher than 0.692 occur (for some species pairs) in every 1 of the 13 studied *Saccharomycetales* genera, and values higher than 0.737 occur in 11 of the 13 genera [40]. Thus, the AAIs of PYCC 10015 and PYCC 10016 to their closest relatives are consistent with AAI levels that correspond to interspecies divergences, in *Saccharomycetales* as well as in *Phaffomycetales*, and support the contention that they represent new species.

## Metabolic profiling

The metabolic profile of PYCC 10015 is consistent with other members of the *Cyberlindnera* genus [5,9,41]. Assimilation of cellobiose and d-xylose suggests an association with rotting wood as expected from the isolation environment [42]. PYCC 10015 can be differentiated from related species *C. galapagoensis* CBS 13997^T^ and *C. qingyuanensis* NYNU 223283^T^ [16,17] in that maltose is weakly assimilated and that inulin, succinate and citrate are not assimilated. PYCC 10015 grows on 10% NaCl, whereas *C. galapagoensis* CBS 13997^T^ shows limited growth and *C. qingyuanensis* NYNU 223283^T^ does not.

The metabolic profile of PYCC 10016 is similar to that of other *Barnettozyma* species [43]. It can be differentiated from closely related species *B. vustinii* CBS 11554^T^ and *B. sucrosica* CBS 11512^T^ [11,44] in that PYCC 10016 cannot ferment glucose, nor can it assimilate l-sorbose, d-xylose or l-rhamnose; ribitol assimilation is weak and lactose can be assimilated (weakly and delayed). It can assimilate cellobiose, salicin, l-lysine and cadaverine, suggesting an association with plant decomposition which is in accord with its environment of isolation [42].

As expected, neither PYCC 10015 nor PYCC 10016 can assimilate methanol [3]. Differentiation from closely related species in carbon and nitrogen source preferences further suggests that these isolates are novel species.

## Taxonomy

### Description of *Barnettozyma discipulorum* A.P. Ryan, C. Carvalho, Y. Zhao, J. Decuseara, M. Osborne, P. Heneghan, K.P. Byrne, K.H. Wolfe, T. Ó Cróinín, J.P. Sampaio **&** G. Butler sp. nov.

**Fig. 2.**
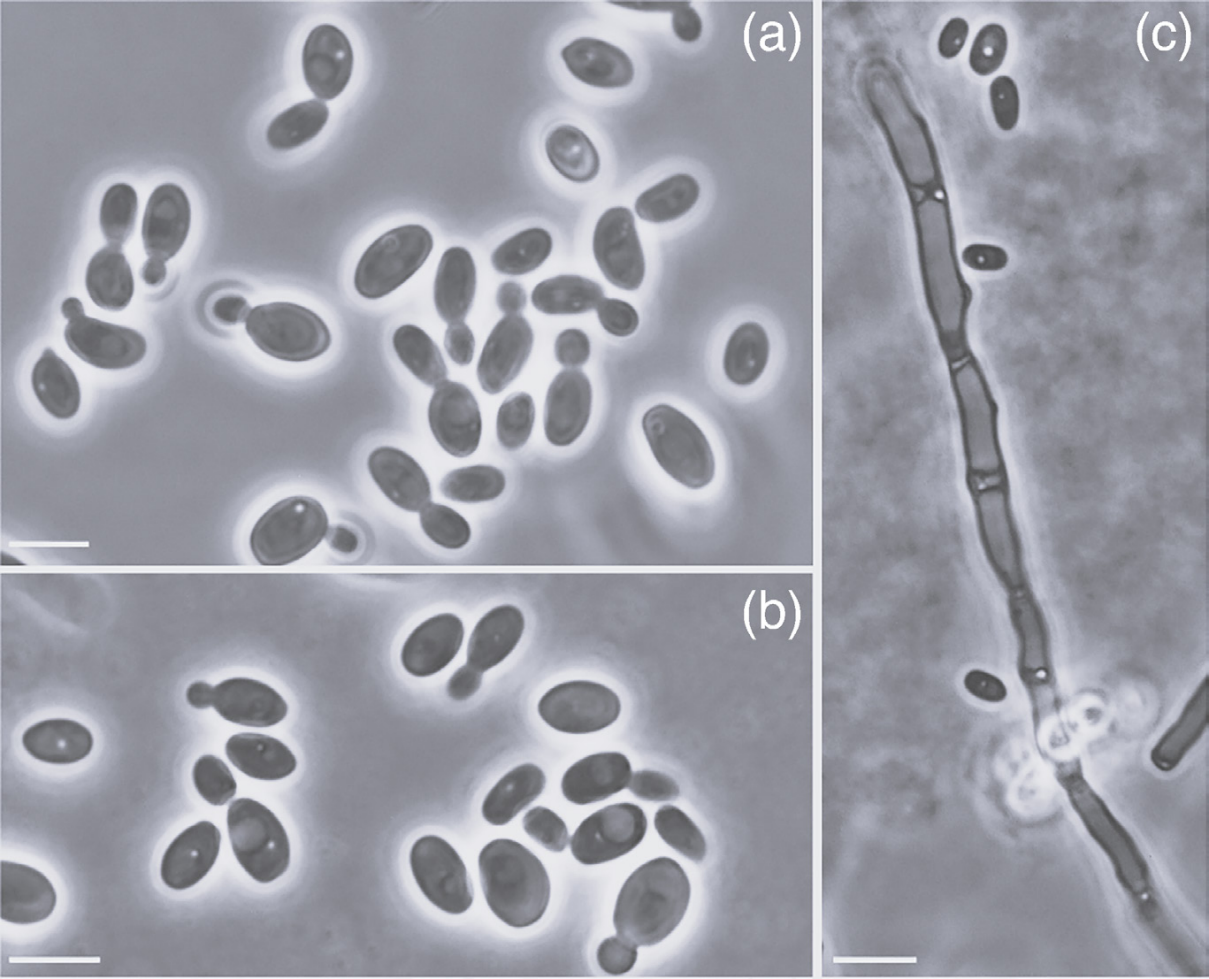
Budding cells of *B. discipulorum* sp. nov. PYCC 10016 (**a**) and *C. hibernica* sp. nov. PYCC 10015 (**b**), grown on YM agar at 25 °C for 2 days, and detail of a hypha of *C. hibernica* PYCC 10015 (**c**) formed on corn meal agar after 2 weeks of incubation at 18 °C. Scale bars=5 µm.

MycoBank MB 856422. Fig. 2a.

*Barnettozyma discipulorum* (dis.ci.pu.lo′rum. L. gen. pl. n. *discipulorum*, of students, named for the undergraduate students integral to its discovery).

Typification: Ireland: County Cork: Glengarriff Woods Nature Reserve, 51.755050, –9.567230, surface soil (3 cm) in a histic horizon, at the base of a mature *Q. petraea* tree in an oak forest, 1 September 2023, G. Butler. The holotype, PYCC 10016-H, is permanently maintained in a metabolically inactive state in the Portuguese Yeast Culture Collection (PYCC), Caparica, Portugal. The ex-holotype cultures are PYCC 10016 (at PYCC), CBS 18649 (at Westerdijk Fungal Biodiversity Institute, Utrecht, The Netherlands) and UCD2008 (at University College Dublin, Ireland). GenBank accession numbers PQ384456 (ITS), PQ373935 (D1/D2 domain of LSU) and JBIEIB000000000 (genome).

Culture characteristics: After 7 days of growth on yeast-malt (YM) agar (0.3% yeast extract, 0.3% malt extract, 0.5% peptone, 1% dextrose and 2% agar) at 25 °C, the streak culture is smooth, cream-colored and butyrous, and colonies have an entire margin. After 3 days of growth on YM agar at 25 °C, cells are ellipsoidal to sub-globose (6–3×4–3 µm) and budding is frequently polar (Fig. 2a). On Dalmau plates after 2 weeks at 18 °C, no pseudohyphae nor true hyphae are formed. Asci and ascospores were not observed after prolonged incubation (2 months) on YM agar, 5% maltose agar, acetate agar and corn meal agar at 18 °C.

Physiological and biochemical characteristics: Glucose is not fermented. Carbon compounds assimilated: d-glucose, cellobiose, salicin, lactose (delayed and weak), glycerol, ribitol (delayed), xylitol (delayed), d-glucitol, d-mannitol, galactitol (delayed), d-glucono-1,5-lactone, dl-lactate, succinate, citrate (delayed), ethanol and l-malic acid. Carbon compounds not assimilated: d-galactose, l-sorbose, d-glucosamine, d-ribose, d-xylose, l-arabinose, d-arabinose, l-rhamnose, sucrose, maltose, *α*,*α*-trehalose, methyl *α*-d-glucoside, melibiose, raffinose, melezitose, inulin, soluble starch, erythritol, myo-inositol, d-gluconate, d-glucuronate, methanol, l-tartaric acid and protocatechuic acid. Nitrogen compounds assimilated: nitrate, nitrite, ethylamine, l-lysine and cadaverine. Nitrogen compounds not assimilated: creatine and creatinine. Growth in the presence of 10% NaCl. No growth in the presence of 0.01% cycloheximide and in the absence of vitamins. Growth is positive but weak at 30 °C and negative at 35 °C. Starch-like compounds are not produced. Hydrolysis of urea and diazonium blue B (DBB) reaction are negative.

### Description of *Cyberlindnera hibernica* A.P. Ryan, C. Carvalho, Y. Zhao, J. Decuseara, M. Osborne, P. Heneghan, K.P. Byrne, K.H. Wolfe, T. Ó Cróinín, J.P. Sampaio & G. Butler sp. nov.

MycoBank MB 856421. Fig. 2(b, c).

*Cyberlindnera hibernica* (hi.ber′ni.ca. L. fem. adj. *hibernica*, of Ireland).

Typification: Ireland: County Cork: Glengarriff Woods Nature Reserve, 51.757080, –9.567610, surface soil (3 cm) in a histic horizon, at the base of a mature *Q. petraea* tree in an oak forest, 1 September 2023, G. Butler. The holotype, PYCC 10015-H, is permanently maintained in a metabolically inactive state in the Portuguese Yeast Culture Collection (PYCC), Caparica, Portugal. The ex-holotype cultures are PYCC 10015 (at PYCC), CBS 18648 (at Westerdijk Fungal Biodiversity Institute, Utrecht, The Netherlands) and UCD1070 (at University College Dublin, Ireland). GenBank accession numbers PQ384455 (ITS), PQ373936 (D1/D2 domain of LSU) and JBIEIC000000000 (genome).

Culture characteristics: After 7 days of growth on YM agar at 25 °C, the streak culture is smooth, cream-colored and butyrous, and colonies have an entire margin. After 3 days of growth on YM agar at 25 °C, cells are ellipsoidal (6–4×4–2 µm) and budding is frequently multilateral (Fig. 2b). On Dalmau plates after 2 weeks at 18 °C, true hyphae are formed, and they grow into the agar (Fig. 2c). Asci and ascospores were not observed after prolonged incubation (2 months) on YM agar, 5% maltose agar, acetate agar and corn meal agar at 18 °C.

Physiological and biochemical characteristics: Glucose and sucrose are fermented. Compounds not fermented: galactose, maltose, methyl *α*-d-glucoside, *α*,*α*-trehalose, melibiose, lactose, cellobiose, melezitose and raffinose. Carbon compounds assimilated: d-glucose, d-xylose, sucrose, maltose (weak), cellobiose, salicin, raffinose, glycerol, xylitol, d-glucitol, d-mannitol, galactitol (delayed), d-gluconate, dl-lactate, ethanol and l-malic acid. Carbon compounds not assimilated: d-galactose, l-sorbose, d-glucosamine, d-ribose, l-arabinose, d-arabinose, l-rhamnose, α,α-trehalose, methyl α-d-glucoside, melibiose, lactose, melezitose, inulin, soluble starch, erythritol, ribitol, myo-inositol, d-glucono-1,5-lactone, d-glucuronate, succinate, citrate, methanol and l-tartaric acid. Nitrogen compounds assimilated: ethylamine, l-lysine and cadaverine. Nitrogen compounds not assimilated: nitrate, nitrite, creatine and cadaverine. Growth in the presence of 10% NaCl. No growth in the presence of 0.01% cycloheximide and in the absence of vitamins. Growth is positive at 25 °C and negative at 30 °C. Starch-like compounds are not produced. Hydrolysis of urea and DBB reaction are negative.

## Supplementary material

10.1099/ijsem.0.006898 Uncited Supplementary Material 1.

## Abbreviations

AAI: average aa identity
ANI: average nt identity
DBB: diazonium blue B
ITS: internal transcribed spacer
ONT: Oxford Nanopore Technologies
SCO: single-copy orthologue
T: ex-type culture
YM: yeast-malt
YPD: yeast-peptone-dextrose
