# Supplementary material for: Cyberlindnera hibernica sp. nov. and Barnettozyma discipulorum sp. nov., isolated from forest soil in Ireland
Source: Int J Syst Evol Microbiol. 2025 Sep 9;75(9):006898. doi: 10.1099/ijsem.0.006898 (PMC12451629; doi:10.1099/ijsem.0.006898)
Supplement: Uncited Supplementary Material 1. [file ijsem-75-06898-s001.pdf]

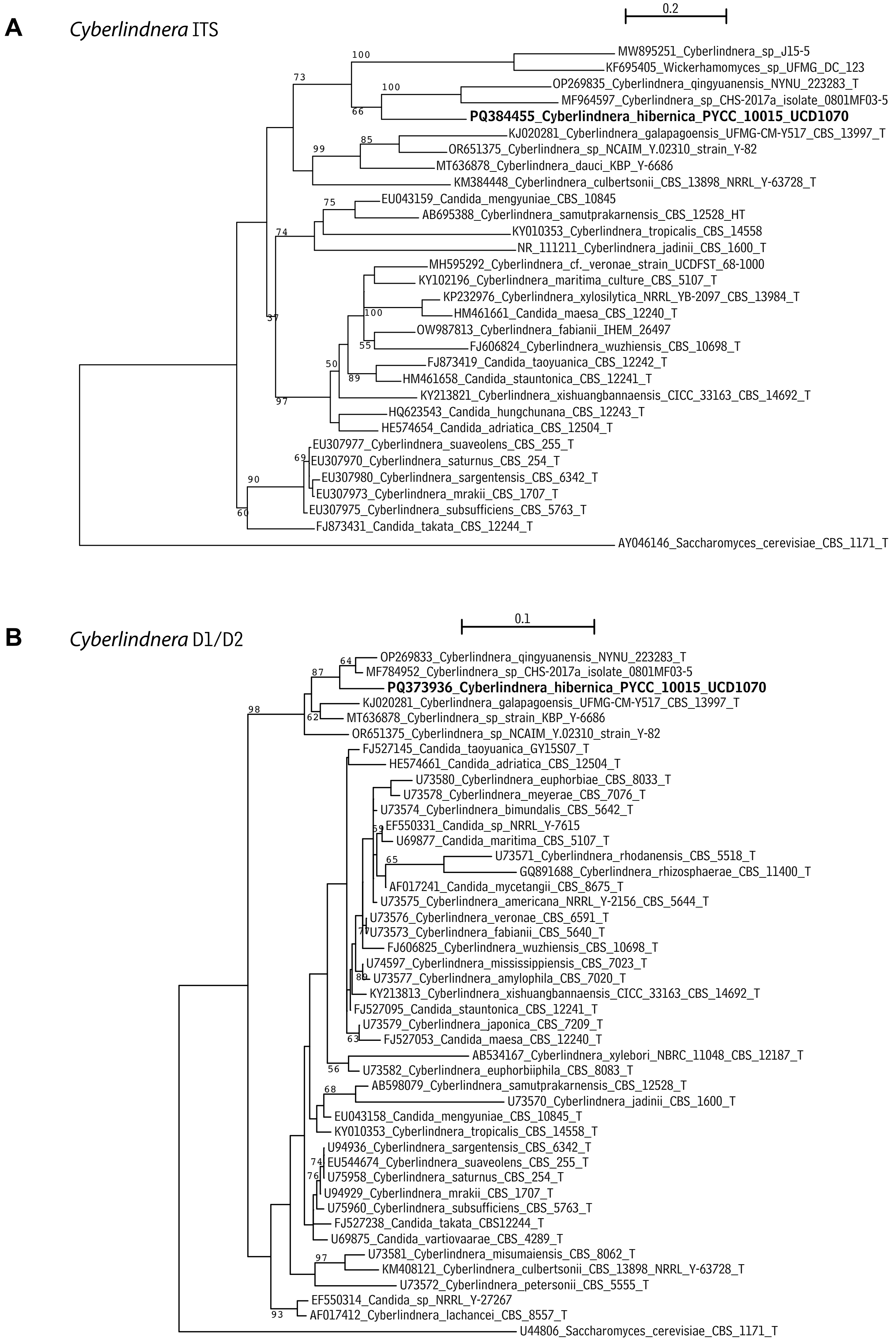

Figure S1. Phylogenetic placement of *Cyberlindnera hibernica* sp. nov. among species of the *Cyberlindnera* clade, based on maximum likelihood analysis of sequences of (A) the ITS region of the rRNA gene array, and (B) the D1/D2 domain of the LSU rRNA gene. NCBI database accession numbers are shown, and the suffix "_T" indicates type strains. Bootstrap support values are shown for branches with >50% support.

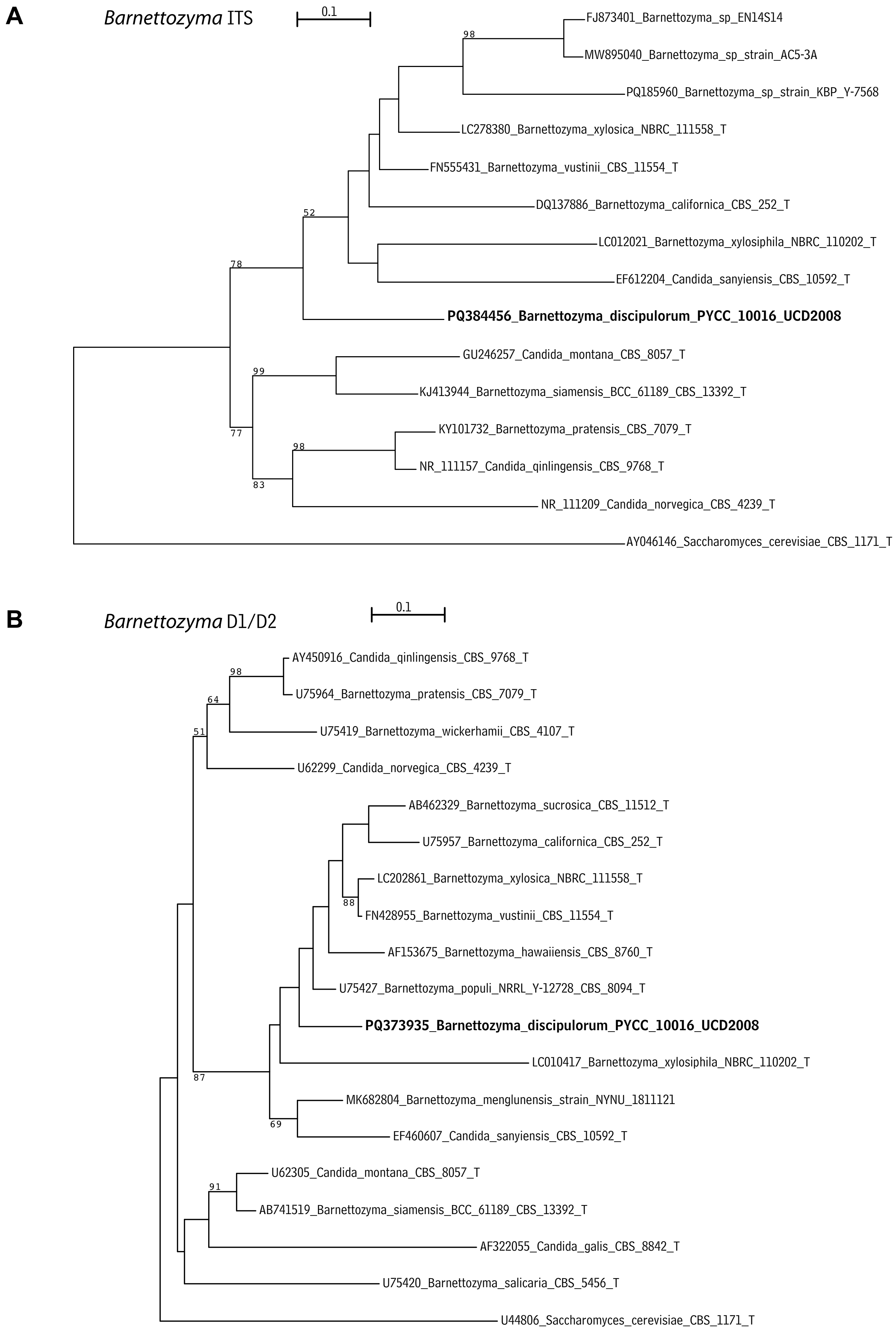

Figure S2. Phylogenetic placement of *Barnettozyma discipulorum* sp. nov. among species of the *Barnettozyma* clade, based on maximum likelihood analysis of sequences of (A) the ITS region of the rRNA gene array, and (B) the D1/D2 domain of the LSU rRNA gene. NCBI database accession numbers are shown, and the suffix "_T" indicates type strains. Bootstrap support values are shown for branches with >50% support.

Table S1. Yeast species isolated from all sampled Irish soils, and from Glengarriff Woods, in 2023. Numbers show the number of independent soil samples from which each species was isolated.

Species	Soil samples from Ireland	Soil samples from Glengarriff Woods
Apiotrichum dulcitum	1	
Apiotrichum porosum	2	
Babjeviella inositovora	1	
Barnettozyma californica	6	
Barnettozyma pratensis	2	
Barnettozyma sp. nov. (*B. discipulorum*)	1	1
Candida oleophila	1	
Candida pseudolambica	1	1
Candida railenensis	2	
Candida sake	4	1
Candida santamariae	1	
Candida solani	1	
Candida sphagnicola	1	
Candida subhashii	1	
Candida vartiovaarae	1	
Clavispora reshetovae	1	
Cutaneotrichosporon moniliiforme	1	
Cyberlindnera sp. nov. (*C. hibernica*)	1	1
Cyberlindnera suaveolens	1	
Cyberlindnera sylvatica	1	
Debaryomyces prosopidis/hansenii	2	
Diutina catenulata	1	
Hanseniaspora menglaensis	1	
Hanseniaspora meyeri	1	
Hanseniaspora osmophila	1	
Hanseniaspora thailandica	1	
Hanseniaspora uvarum	4	
Hanseniaspora valbyensis	2	2
Kazachstania servazzii	12	
Kazachstania telluris	4	
Kluyveromyces dobzhanski	1	
Kluyveromyces marxianus	7	
Nadsonia starkey-henricii	1	
Pichia nakasei	1	
Saccharomyces eubayanus	5	
Saccharomyces kudriavzevii	1	
Saccharomyces paradoxus	14	3
Saccharomyces uvarum	1	1
Saitozyma podzolica	1	
Torulaspora delbrueckii	3	1
Trichosporon middelhovenii	1	
Trichosporon porosum	5	1
Vanrija pseudolonga	1	
Wickerhamomyces anomalus	4	
Total	106	12

Table S2. ANI and AAI values between genomes in the *Cyberlindnera* clade. Strain numbers and NCBI accession numbers are as shown in Fig. 1.

ANI (Average Nucleotide Identity) values in the *Cyberlindnera* clade, calculated using OrthoANI.

	Cyberlindnera hibernica PYCC 10015	*Cyberlindnera galapagoensis*	*Cyberlindnera subsufficiens*	*Cyberlindnera sargentensis*	*Cyberlindnera mrakii*	*Cyberlindnera suaveolens*	*Cyberlindnera saturnus*	*Candida sp. NRRL YB-4088*	*Cyberlindnera sp. NRRL Y-27103*	*Cyberlindnera maclurae*	*Cyberlindnera misumaiensis*	*Candida vartiovaarae*	*Candida takata*	*Cyberlindnera samutprakarnensis*	*Cyberlindnera jadinii*	*Cyberlindnera lachancei*	*Cyberlindnera sp. NRRL Y-27267*	*Cyberlindnera americana*	*Cyberlindnera xylosilytica*
Cyberlindnera hibernica PYCC 10015		0.732	0.713	0.710	0.712	0.715	0.714	0.711	0.705	0.706	0.708	0.716	0.715	0.705	0.707	0.706	0.708	0.701	0.710
Cyberlindnera galapagoensis	0.732		0.714	0.711	0.712	0.716	0.715	0.709	0.701	0.699	0.702	0.711	0.712	0.706	0.705	0.711	0.712	0.700	0.698
Cyberlindnera subsufficiens	0.713	0.714		0.838	0.846	0.867	0.861	0.720	0.714	0.711	0.714	0.728	0.730	0.722	0.721	0.726	0.727	0.705	0.701
Cyberlindnera sargentensis	0.710	0.711	0.838		0.891	0.864	0.859	0.716	0.711	0.708	0.711	0.723	0.727	0.719	0.718	0.721	0.724	0.701	0.698
Cyberlindnera mrakii	0.712	0.712	0.846	0.891		0.873	0.871	0.717	0.713	0.710	0.713	0.725	0.729	0.720	0.719	0.724	0.725	0.703	0.701
Cyberlindnera suaveolens	0.715	0.716	0.867	0.864	0.873		0.924	0.722	0.717	0.714	0.716	0.731	0.734	0.723	0.722	0.726	0.729	0.705	0.703
Cyberlindnera saturnus	0.714	0.715	0.861	0.859	0.871	0.924		0.721	0.716	0.712	0.716	0.730	0.732	0.722	0.722	0.725	0.728	0.705	0.703
Candida sp. NRRL YB-4088	0.711	0.709	0.720	0.716	0.717	0.722	0.721		0.770	0.752	0.761	0.721	0.721	0.712	0.713	0.715	0.717	0.700	0.701
Cyberlindnera sp. NRRL Y-27103	0.705	0.701	0.714	0.711	0.713	0.717	0.716	0.770		0.788	0.811	0.718	0.717	0.708	0.708	0.710	0.711	0.694	0.698
Cyberlindnera maclurae	0.706	0.699	0.711	0.708	0.710	0.714	0.712	0.752	0.788		0.809	0.717	0.717	0.704	0.708	0.705	0.707	0.693	0.702
Cyberlindnera misumaiensis	0.708	0.702	0.714	0.711	0.713	0.716	0.716	0.761	0.811	0.809		0.720	0.719	0.707	0.710	0.708	0.710	0.694	0.701
Candida vartiovaarae	0.716	0.711	0.728	0.723	0.725	0.731	0.730	0.721	0.718	0.717	0.720		0.762	0.720	0.722	0.721	0.723	0.703	0.707
Candida takata	0.715	0.712	0.730	0.727	0.729	0.734	0.732	0.721	0.717	0.717	0.719	0.762		0.723	0.723	0.723	0.726	0.704	0.706
Cyberlindnera samutprakarnensis	0.705	0.706	0.722	0.719	0.720	0.723	0.722	0.712	0.708	0.704	0.707	0.720	0.723		0.846	0.721	0.723	0.699	0.696
Cyberlindnera jadinii	0.707	0.705	0.721	0.718	0.719	0.722	0.722	0.713	0.708	0.708	0.710	0.722	0.723	0.846		0.718	0.719	0.699	0.701
Cyberlindnera lachancei	0.706	0.711	0.726	0.721	0.724	0.726	0.725	0.715	0.710	0.705	0.708	0.721	0.723	0.721	0.718		0.837	0.701	0.693
Cyberlindnera sp. NRRL Y-27267	0.708	0.712	0.727	0.724	0.725	0.729	0.728	0.717	0.711	0.707	0.710	0.723	0.726	0.723	0.719	0.837		0.703	0.693
Cyberlindnera americana	0.701	0.700	0.705	0.701	0.703	0.705	0.705	0.700	0.694	0.693	0.694	0.703	0.704	0.699	0.699	0.701	0.703		0.724
Cyberlindnera xylosilytica	0.710	0.698	0.701	0.698	0.701	0.703	0.703	0.701	0.698	0.702	0.701	0.707	0.706	0.696	0.701	0.693	0.693	0.724	

MEAN:	0.726
STDEV:	0.042
MAX:	0.924
MIN:	0.693

AAI (Average Aminoacid Identity) values in the *Cyberlindnera* clade, calculated using CompareM.

	Cyberlindnera hibernica PYCC 10015	*Cyberlindnera galapagoensis*	*Cyberlindnera subsufficiens*	*Cyberlindnera sargentensis*	*Cyberlindnera mrakii*	*Cyberlindnera suaveolens*	*Cyberlindnera saturnus*	*Candida sp. NRRL YB-4088*	*Cyberlindnera sp. NRRL Y-27103*	*Cyberlindnera maclurae*	*Cyberlindnera misumaiensis*	*Candida vartiovaarae*	*Candida takata*	*Cyberlindnera samutprakarnensis*	*Cyberlindnera jadinii*	*Cyberlindnera lachancei*	*Cyberlindnera sp. NRRL Y-27267*	*Cyberlindnera americana*	*Cyberlindnera xylosilytica*
Cyberlindnera hibernica PYCC 10015		0.692	0.669	0.666	0.668	0.670	0.669	0.651	0.652	0.652	0.656	0.656	0.658	0.647	0.648	0.644	0.642	0.635	0.639
Cyberlindnera galapagoensis	0.692		0.659	0.657	0.658	0.660	0.659	0.642	0.643	0.643	0.646	0.647	0.650	0.642	0.644	0.639	0.637	0.628	0.631
Cyberlindnera subsufficiens	0.669	0.659		0.875	0.886	0.900	0.898	0.694	0.702	0.702	0.708	0.713	0.717	0.695	0.700	0.686	0.681	0.652	0.656
Cyberlindnera sargentensis	0.666	0.657	0.875		0.916	0.892	0.890	0.689	0.698	0.695	0.703	0.707	0.711	0.690	0.693	0.681	0.677	0.649	0.653
Cyberlindnera mrakii	0.668	0.658	0.886	0.916		0.904	0.902	0.692	0.700	0.698	0.706	0.710	0.714	0.693	0.697	0.683	0.680	0.651	0.653
Cyberlindnera suaveolens	0.670	0.660	0.900	0.892	0.904		0.933	0.694	0.703	0.701	0.708	0.713	0.719	0.695	0.699	0.686	0.683	0.653	0.654
Cyberlindnera saturnus	0.669	0.659	0.898	0.890	0.902	0.933		0.694	0.702	0.701	0.707	0.713	0.716	0.695	0.700	0.685	0.680	0.652	0.654
Candida sp. NRRL YB-4088	0.651	0.642	0.694	0.689	0.692	0.694	0.694		0.808	0.779	0.795	0.684	0.687	0.671	0.674	0.665	0.663	0.635	0.638
Cyberlindnera sp. NRRL Y-27103	0.652	0.643	0.702	0.698	0.700	0.703	0.702	0.808		0.825	0.853	0.690	0.692	0.675	0.677	0.667	0.666	0.637	0.641
Cyberlindnera maclurae	0.652	0.643	0.702	0.695	0.698	0.701	0.701	0.779	0.825		0.844	0.690	0.693	0.675	0.677	0.667	0.665	0.637	0.638
Cyberlindnera misumaiensis	0.656	0.646	0.708	0.703	0.706	0.708	0.707	0.795	0.853	0.844		0.695	0.698	0.679	0.683	0.671	0.669	0.640	0.642
Candida vartiovaarae	0.656	0.647	0.713	0.707	0.710	0.713	0.713	0.684	0.690	0.690	0.695		0.773	0.689	0.693	0.680	0.678	0.640	0.642
Candida takata	0.658	0.650	0.717	0.711	0.714	0.719	0.716	0.687	0.692	0.693	0.698	0.773		0.693	0.697	0.684	0.682	0.643	0.645
Cyberlindnera samutprakarnensis	0.647	0.642	0.695	0.690	0.693	0.695	0.695	0.671	0.675	0.675	0.679	0.689	0.693		0.894	0.672	0.669	0.636	0.638
Cyberlindnera jadinii	0.648	0.644	0.700	0.693	0.697	0.699	0.700	0.674	0.677	0.677	0.683	0.693	0.697	0.894		0.674	0.671	0.638	0.641
Cyberlindnera lachancei	0.644	0.639	0.686	0.681	0.683	0.686	0.685	0.665	0.667	0.667	0.671	0.680	0.684	0.672	0.674		0.857	0.634	0.634
Cyberlindnera sp. NRRL Y-27267	0.642	0.637	0.681	0.677	0.680	0.683	0.680	0.663	0.666	0.665	0.669	0.678	0.682	0.669	0.671	0.857		0.632	0.634
Cyberlindnera americana	0.635	0.628	0.652	0.649	0.651	0.653	0.652	0.635	0.637	0.637	0.640	0.640	0.643	0.636	0.638	0.634	0.632		0.713
Cyberlindnera xylosilytica	0.639	0.631	0.656	0.653	0.653	0.654	0.654	0.638	0.641	0.638	0.642	0.642	0.645	0.638	0.641	0.634	0.634	0.713	

MEAN:	0.694
STDEV:	0.067
MAX:	0.933
MIN:	0.628

Table S3. ANI and AAI values between genomes in the *Barnettozyma* clade. Strain numbers and NCBI accession numbers are as shown in Fig. 1.

ANI (Average Nucleotide Identity) values in the *Barnettozyma* clade, calculated using OrthoANI.

	Barnettozyma discipulorum PYCC 10016	*Barnettozyma populi*	*Barnettozyma sucrosica*	*Barnettozyma vustinii*	*Barnettozyma californica*	*Barnettozyma hawaiiensis*	*Barnettozyma sp. yHDO593*	*Candida sanyiensis*	*Barnettozyma pratensis*	*Barnettozyma salicaria*	*Barnettozyma botsteinii*	*Phaffomyces opuntiae*
Barnettozyma discipulorum PYCC 10016		0.739	0.737	0.746	0.739	0.739	0.718	0.729	0.719	0.706	0.715	0.702
Barnettozyma populi	0.739		0.817	0.832	0.801	0.804	0.737	0.736	0.718	0.707	0.712	0.700
Barnettozyma sucrosica	0.737	0.817		0.823	0.796	0.799	0.736	0.735	0.717	0.706	0.711	0.700
Barnettozyma vustinii	0.746	0.832	0.823		0.840	0.838	0.743	0.743	0.724	0.710	0.715	0.703
Barnettozyma californica	0.739	0.801	0.796	0.840		0.806	0.737	0.733	0.721	0.707	0.715	0.704
Barnettozyma hawaiiensis	0.739	0.804	0.799	0.838	0.806		0.737	0.738	0.716	0.706	0.710	0.699
Barnettozyma sp. yHDO593	0.718	0.737	0.736	0.743	0.737	0.737		0.718	0.707	0.702	0.705	0.697
Candida sanyiensis	0.729	0.736	0.735	0.743	0.733	0.738	0.718		0.700	0.694	0.695	0.680
Barnettozyma pratensis	0.719	0.718	0.717	0.724	0.721	0.716	0.707	0.700		0.729	0.755	0.747
Barnettozyma salicaria	0.706	0.707	0.706	0.710	0.707	0.706	0.702	0.694	0.729		0.727	0.719
Barnettozyma botsteinii	0.715	0.712	0.711	0.715	0.715	0.710	0.705	0.695	0.755	0.727		0.763
Phaffomyces opuntiae	0.702	0.700	0.700	0.703	0.704	0.699	0.697	0.680	0.747	0.719	0.763	

MEAN:	0.734
STDEV:	0.039
MAX:	0.840
MIN:	0.680

AAI (Average Aminoacid Identity) values in the *Barnettozyma* clade, calculated using CompareM.

	Barnettozyma discipulorum PYCC 10016	*Barnettozyma populi*	*Barnettozyma sucrosica*	*Barnettozyma vustinii*	*Barnettozyma californica*	*Barnettozyma hawaiiensis*	*Barnettozyma sp. yHDO593*	*Candida sanyiensis*	*Barnettozyma pratensis*	*Barnettozyma salicaria*	*Barnettozyma botsteinii*	*Phaffomyces opuntiae*
Barnettozyma discipulorum		0.730	0.728	0.737	0.731	0.721	0.692	0.713	0.685	0.668	0.676	0.655
Barnettozyma populi	0.730		0.844	0.863	0.839	0.813	0.737	0.748	0.694	0.675	0.684	0.662
Barnettozyma sucrosica	0.728	0.844		0.854	0.833	0.808	0.736	0.746	0.692	0.673	0.682	0.661
Barnettozyma vustinii	0.737	0.863	0.854		0.872	0.838	0.747	0.756	0.701	0.680	0.690	0.665
Barnettozyma californica	0.731	0.839	0.833	0.872		0.818	0.739	0.748	0.695	0.677	0.685	0.662
Barnettozyma hawaiiensis	0.721	0.813	0.808	0.838	0.818		0.731	0.736	0.687	0.669	0.678	0.657
Barnettozyma sp. yHDO593	0.692	0.737	0.736	0.747	0.739	0.731		0.703	0.666	0.656	0.660	0.644
Candida sanyiensis	0.713	0.748	0.746	0.756	0.748	0.736	0.703		0.680	0.665	0.673	0.652
Barnettozyma pratensis	0.685	0.694	0.692	0.701	0.695	0.687	0.666	0.680		0.705	0.719	0.686
Barnettozyma salicaria	0.668	0.675	0.673	0.680	0.677	0.669	0.656	0.665	0.705		0.695	0.669
Barnettozyma botsteinii	0.676	0.684	0.682	0.690	0.685	0.678	0.660	0.673	0.719	0.695		0.717
Phaffomyces opuntiae	0.655	0.662	0.661	0.665	0.662	0.657	0.644	0.652	0.686	0.669	0.717	

MEAN:	0.717
STDEV:	0.059
MAX:	0.872
MIN:	0.644
